# Fractional quantum oscillator and disorder in the vibrational spectra

**DOI:** 10.1038/s41598-022-16597-2

**Published:** 2022-07-22

**Authors:** V. A. Stephanovich, E. V. Kirichenko, V. K. Dugaev, Jackie Harjani Sauco, Belén López Brito

**Affiliations:** 1 Institute of Physics, University of Opole, ul. Oleska 48, 45-052 Opole, Poland; 2 Department of Physics and Medical Engineering, Rzeszów University of Technology, al. Powstańców Warszawy 6, 35-959 Rzeszów, Poland; 3 Department of Mathematics, Universidad de Las Palmas de Gran Canaria, Campus de Tafira Baja, C.P. 35017 Las Palmas, Spain

**Keywords:** Theoretical physics, Structure of solids and liquids

## Abstract

We study the role of disorder in the vibration spectra of molecules and atoms in solids. This disorder may be described phenomenologically by a fractional generalization of ordinary quantum-mechanical oscillator problem. To be specific, this is accomplished by the introduction of a so-called fractional Laplacian (Riesz fractional derivative) to the Scrödinger equation with three-dimensional (3D) quadratic potential. To solve the obtained 3D spectral problem, we pass to the momentum space, where the problem simplifies greatly as fractional Laplacian becomes simply $k^\mu$, *k* is a modulus of the momentum vector and $\mu$ is Lévy index, characterizing the degree of disorder. In this case, $\mu \rightarrow 0$ corresponds to the strongest disorder, while $\mu \rightarrow 2$ to the weakest so that the case $\mu =2$ corresponds to “ordinary” (i.e. that without fractional derivatives) 3D quantum harmonic oscillator. Combining analytical (variational) and numerical methods, we have shown that in the fractional (disordered) 3D oscillator problem, the famous orbital momentum degeneracy is lifted so that its energy starts to depend on orbital quantum number *l*. These features can have a strong impact on the physical properties of many solids, ranging from multiferroics to oxide heterostructures, which, in turn, are usable in modern microelectronic devices.

## Introduction

Although disorder (especially strong like substance amorphization) in a solid is usually considered to be a trouble for its possible device applications, its constructive properties have become increasingly clear in recent years as they give an additional possibility to fine tune their physical properties. Specifically, by varying the type and concentration of different imperfections (like point or extended defects), we can customize the characteristics of such material to meet specific requirements, needed, for instance, in electronics. Disordered semiconductors and dielectrics like organometallic halide perovskites^1,2^ are widely used in photovoltaic cells, light-emitting devices, and nanolasers^3,4^. The above disorder influences phonon and electron spectra of a substance, leading to the distribution of the internal magnetic, electric and elastic fields. This is the case for so-called multiferroics, where ferroelectric and magnetic orders coexist^5^. It is well-known (see, e.g.^6,7^) that strong disorder is well described by Non-Gaussian probability distributions, having so-called long tails. In other words, these distributions decay at infinities (sometimes much) slower, that Gaussian one. The most prominent example here is so-called Lévy flights^8–13^, which are Markovian random processes whose probability density functions (pdf) are Lévy stable laws, characterized by the Lévy index $0<\mu \le 2$. In the infinite space, it is profitable to define the corresponding pdf in terms of its characteristic function, i.e. Fourier transform. The pdf of a typical Lévy flight is usually determined by the fractional Fokker–Planck (FP) Eq. (^8^). In dimensionless units (diffusion coefficient and particle mass are set to unity) it reads

1 $$\begin{aligned} \partial _t f(\mathbf{r},t)=-|\Delta |^{\mu /2}f(\mathbf{r},t)+\nabla \bigg [\nabla U(\mathbf{r})f(\mathbf{r},t)\bigg ] \end{aligned}$$

where $|\Delta |^{\mu /2}$ is an *n*-dimensional fractional Laplacian (Riesz fractional derivative^6,7^)

2 $$\begin{aligned}-|\Delta |^{\mu /2}f(\mathbf{x})=A_{n,\mu } \int \frac{f(\mathbf{u})-f(\mathbf{x})}{|\mathbf{u}-\mathbf{x}|^{\mu +n}}d^nu, \end{aligned}$$

3 $$\begin{aligned}A_{n,\mu }=\frac{2^\mu \Gamma \left( \frac{\mu +n}{2}\right) }{\pi ^{n/2}|\Gamma (-\mu /2)|}, \end{aligned}$$

which at $\mu =2$ yields the ordinary one^6,7^. Here $\Gamma (x)$ is $\Gamma$-function^14^, $\nabla$ is (also *n*- dimensional) gradient operator and $U(\mathbf{r})$ is an external potential, in which the anomalous diffusion occurs^15^. The operator (Eq. 2) is spatially nonlocal, which is a source of memory effects in strongly disordered systems.

It had been shown by Laskin, that the substitution of the Gaussian measure by the Lévy one in the Feynman path integrals generates so-called fractional quantum mechanics^16,17^, where an ordinary Laplacian in the Schrödinger equation is substituted with fractional one (Eq. 2). In context of the above disordered solids, it is important to consider the influence of disorder onto the spectra of their collective atomic vibrations, which constitute well-known phonons in an ordered substance. As the latter vibrations are described by the quantum harmonic oscillator model, it is reasonable to consider the fractional analog of real 3D quantum oscillator problem.

In the present paper we shall find the spectrum of 3D fractional oscillator for arbitrary index $0<\mu \le 2$. As this problem resides on the whole 3D space, we shall solve it using Fourier transformation to the momentum space. In latter space the problem becomes local as the Fourier image of the operator (Eq. 2) is simply $k^\mu$ ($k\equiv |\mathbf{k}|$, where $\mathbf{k}$ is the momentum vector), while the oscillator potential gives the ordinary Laplacian in the momentum space. In other words, the initial fractional Schrödinger equation gives ordinary one in $\mathbf{k}$ space. The only difference is that the potential in this new equation will be $k^\mu$. This implies that to solve our problem in momentum space, we can use well developed numerical and variational techniques.

## The spectral problem for fractional quantum harmonic oscillator

In the units, where $\hbar =m=\omega =1$ (*m* and $\omega$ are the mass and frequency of oscillating particle respectively), the spectral problem for 3D fractional oscillator reads

4 $$\begin{aligned} -|\Delta |^{\mu /2}\psi _{nlm\mu }(\mathbf{r})+(x^{2}+y^{2}+z^{2}) \psi _{nlm\mu }(\mathbf{r})=2E_{nl\mu }\psi _{nlm\mu }(\mathbf{r}), \end{aligned}$$

where the operator $|\Delta |^{\mu /2}$ is defined by the expression (2) and $\psi _{nlm\mu }(x)$ is the eigenfunction of a fractional quantum harmonic oscillator having the eigenenergy $E_{nl\mu }$ for any specific $\mu$ value. In general 3D case, as it is typical for the quantum-mechanical problems in central force potentials, the eigenfunction $\psi$ and eigenenergy *E* of the problem (4) depend on three quantum numbers *n*, *l*, *m* which are principal, orbital and magnetic quantum numbers respectively^18,19^.

As we discussed above, the most profitable method to solve this problem is to pass to the momentum space, which permits to reduce the integral equation (4) to the differential one. Namely, as it is well-known that the combinations $x^2\psi (x,y,z)$, $y^2\psi (x,y,z)$ and $z^2\psi (x,y,z)$ (here we suppress the indices $nlm\mu$ for brevity) in momentum space render as second derivatives $\frac{\partial ^2}{\partial k_x^2}\psi (k_x,k_y,k_z)$, $\frac{\partial ^2}{\partial k_y^2}\psi (k_x,k_y,k_z)$ and $\frac{\partial ^2}{\partial k_z^2}\psi (k_x,k_y,k_z)$, the potential term in the equation (4) renders simply as an ordinary Laplacian $\Delta _\mathbf{k}\equiv \frac{\partial ^2}{\partial k_x^2}+\frac{\partial ^2}{\partial k_y^2}+\frac{\partial ^2}{\partial k_z^2}$ in momentum space. At the same time, the fractional Laplacian in momentum space becomes simply $k^\mu$, where $k\equiv =\sqrt{k_x^2+k_y^2+k_z^2}$ is a vector $\mathbf{k}$ modulus. Latter definition immediately implies that $0\le k< \infty$. This means that in momentum space, the equation (4) renders as

5 $$\begin{aligned} {{\mathcal H}}_\mathbf{k}\psi _{nlm\mu }(\mathbf{k})= E_{nl\mu }\psi _{nlm\mu }(\mathbf{k}), \ {\mathcal H}_\mathbf{k}= -\Delta _\mathbf{k} +k^\mu , \end{aligned}$$

where ${\mathcal H}_\mathbf{k}$ is the system Hamiltonian in momentum space. One note is in place here. Namely, the equation (5) shows that at $\mu <2$, the famous orbital 3D oscillator degeneracy (see Ref.^18^ for details) is lifted so that the eigenenergy *E* starts to depend on the orbital quantum number *l*. At the same time, as in the fractional case of $\mu <2$, the time-reversal symmetry remains (it is lifted, for instance, in the external magnetic field^18^), we still have degeneracy with respect to the quantum number *m*. Namely, the spectrum is invariant as $m \leftrightarrow -m$. That is why, in equation (5), we put index *m* explicitly in the wave functions, which are different for different *m*, although the energy *E* does not depend on *m*.

As for any central potential the wave functions are invariant with respect to rotation around the origin^18,19^, this is also the case for our 3D fractional oscillator. This implies that the wave functions $\psi _{nlm\mu }(\mathbf{k})$ admit the separation of angular and radial (in $\mathbf{k}$ space) variables in the form

6 $$\begin{aligned} \psi _{nlm\mu }(\mathbf{k})=R_{nlm\mu }(k)Y_{lm}(\theta , \varphi ), \end{aligned}$$

where $Y_{lm}(\theta , \varphi )=A_{lm}P_l^{|m|}(\cos \theta )e^{im\varphi }$ are spherical harmonics^14,18^. Here $P_l^{|m|}(\cos \theta )$ are associate Legendre polynomials of the first kind and $A_{lm}$ is the corresponding normalization coefficient, which is obtained from the following normalization condition^14,18^

7 $$\begin{aligned} \int _\Omega Y_{lm}^*(\theta , \varphi ) Y_{lm}(\theta , \varphi )d\Omega =1, \end{aligned}$$

where $$d\Omega =\sin \theta d\theta d\varphi $$.

It is well-known from “ordinary” quantum mechanics^18,19^ that 3D quantum oscillator problem for $\mu =2$ admits the factorization of the wave function $\psi (x,y,z)=\psi _x(x)\psi _y(y)\psi _z(z)$ (here we also suppress the lower indices for a moment), which permits to reduce the problem to the three equations for 1D oscillator, containing three different energies, say $E_x$, $E_y$ and $E_z$. In this case, the initial energy $E=E_x+E_y+E_z$. After problem being in such a way factorized, the eigenenergy can be derived in the well-known form^18,19^

8 $$\begin{aligned} E_{\mu =2}=n+\frac{3}{2}, \end{aligned}$$

where $n=n_x+n_y+n_z$ with $n_{x,y,z}$ being the quantum numbers, corresponding to $E_{x,y,z}$ respectively. Such factorization in the “ordinary” case $\mu =2$ reduces the problem of the 3D oscillator to the combinatorial problem with respect to its 1D “components”. In other words, the quantum numbers *nlm* of the resulting 3D problem comprise different combinations (obeying certain combinatorial rules, see Refs.^18,19^ for details) of the initial $n_x$, $n_y$ and $n_z$ numbers.

It had been shown in^19^, that the possibility of such factorization at $\mu =2$ is based on additivity of *both* Laplacian operator (kinetic energy) and the potential $\sim (x^2+y^2+z^2)$. At the same time, in $\mathbf{r}$ space (Eq.4), the fractional Laplacian (Eq. 2) is not additive. That is to say, it does not consist of a sum of the terms, containing separately *x*, *y* and *z* variables. This fact is “transferred” to $\mathbf{k}$ space, where the potential $k^\mu \equiv (k_x^2+k_y^2+k_z^2)^{\mu /2}$ is factorable for $\mu =2$ (ordinary case) only.

This signifies the difference between fractional $\mu <2$ and ordinary $\mu =2$ 3D oscillator problems. This difference implies that for our fractional case we should proceed directly in 3D case. This, however, does not give an essential complication as problem still reduces to 1D one. Namely, after the decomposition (Eq. 6), we obtain following fractional 1D Scrödinger equation for the radial part $R_{nlm\mu }(k)$

9 $$\begin{aligned} \frac{d^2R}{dk^2}+\frac{2}{k} \frac{dR}{dk}+\left[ 2E-\frac{l(l+1)}{k^2}-k^\mu \right] R=0. \end{aligned}$$

Here we also suppress the indices $nlm\mu$ in the functions *R* and energy *E* for a moment. In the quantum mechanics of the central force systems, it is also customary (see, e.g.^18,19^) to pass to the function

10 $$\begin{aligned} \chi (k)=kR(k) \end{aligned}$$

for which the Eq. (9) assumes particularly simple form

11 $$\begin{aligned} \frac{d^2\chi }{dk^2}+\left[ 2E-\frac{l(l+1)}{k^2}-k^\mu \right] \chi =0, \end{aligned}$$

which resembles that for 1D system. This form will be used below to construct trial functions for variational method as well as for numerical calculations, where the form (Eq. 11) permits to avoid divergencies at $k=0$. We note here, that if for the states with $l=0$, the function *R*(*k*) has a maximum at $k=0$, the corresponding function $\chi$ is zero by obvious reason.

As we mentioned above, the Eq. (11) can be readily solved numerically. On the other hand, even approximate analytical solution like variational one is much more desired as it can represent the useful orthogonal set, which can be further employed for the solutions of the problems, not obligatory dealing with fractional quantum mechanics. For instance, it could be well used to look for the solutions of the fractional FP equation (1) in the from of the expansion over the infinite sets of orthogonal functions. That is why we will solve the radial equation (11) variationally and confirm our results by direct numerical solution.

**Figure 1:**
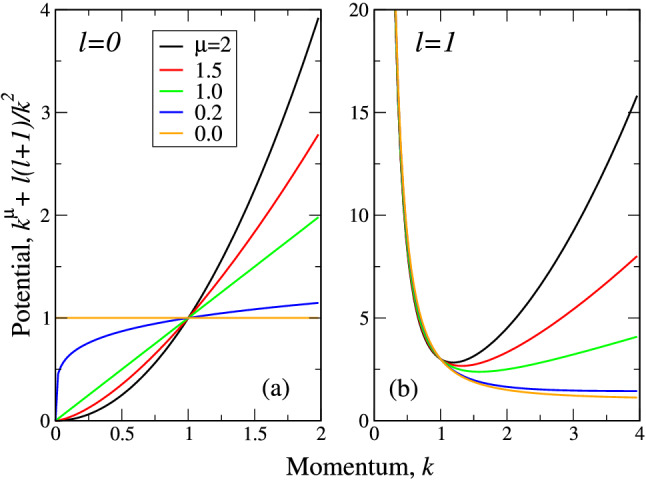
Potential in the Schrödinger equation (9) at $l=0$ (**a**) and $l=1$ (**b**). Lévy indices $\mu$ are coded by colors (legend in (**a**)). Mind the different vertical scales in (**a,b**).

The Eqs. (9), (11) represent Schrödinger equation with the effective 1D potential $|k|^\mu +l(l+1)/k^2$. The plots of this potential at different $\mu$ and *l* are shown in Fig.1. It is seen from Fig.1a that for $l=0$ the problem is identical to that for 1D fractional oscillator^20^, while for $l \ne 0$ (Fig.1b), the centrifugal force drives it outwards. The only difference with the case of 1D oscillator is that now the problem is defined on positive semi-axis $0<k<\infty$ as *k* is now the modulus of a momentum vector. As it is seen from Fig.1b, the centrifugal term in the potential $l(l+1)/k^2$ is dominant at $k \rightarrow 0$, while at $k \rightarrow \infty$ the main contribution comes from $k^\mu$. This means that the whole spectrum of 3D fractional oscillator remains to be discrete similar to the “ordinary” case $\mu =2$. Note that at small $\mu =0.2$ the potential begins to grow at very large *k*, while at $\mu =0$ the potential goes to 1 at $k \rightarrow \infty$. The same situation at $l=0$ gives constant value one for $\mu =0$, see Fig. 1a. Our numerical analysis shows that in 3D case despite the contribution of centrifugal term, at $\mu =0$, the whole oscillator spectrum collapses into one value, see below. This property is similar to that in 1D case^20^. Note that at $l=0$ and $\mu <1$ the potential at $k \rightarrow 0$ the potential has nonanalytical behavior with infinite first derivative. At $l \ne 0$ this asymptotics is “killed” by the centrifugal term.

## Variational solution. Classification of the states of 3D fractional oscillator

It is well-known that the variational principle of quantum mechanics^18^ permits to find the approximate analytical expressions for the wave functions and eigenenergies of corresponding Schrödinger equations. This is especially true for the states of discrete spectrum, which are spatially localized. As usually, the “correct” (i.e. that which minimizes corresponding energy functional with minimal number of variational parameters) set of trial functions should be obtained on the class of functions, having the asymptotics, dictated by the corresponding differential equation.

Note that for the above variational principle to work, it is sufficient even to use the trial functions obeying the correct boundary conditions rather than have the “prescribed” asymptotics. In our case, this means that the trial functions have to be zero at $k \rightarrow \infty$. Such “simple” trial functions can be constructed on the base of the Gaussian one, inherent in an “ordinary” (i.e. that at $\mu =2$) 3D oscillator. In this case, the whole variational spectrum can be thought of in the form of “Hermite polynomials with unknown coefficients”, where the latter coefficients are to be found from the orthogonality conditions, see below. In the next section, we shall compare the relative errors of the variational approaches, based on “fractional” (i.e. with predefined asymptotics) and “Gaussian” trial functions.

To construct the “fractional” trial functions, we should analyze the large *k* asymptotics of the $\chi (k)$, given by the radial (in $\mathbf{k}$ space) Schrödinger equation (11). For that we observe that at large *k* we can neglect all the terms in the square brackets in Eq. (11) except $k^\mu$. This yields the equation for large *k* in the form $d^2\chi (k)/dk^2=k^\mu \chi$. The spatially decaying solution to this equation is proportional to $k^{1/2}K_\nu (u)$, where $\nu =1/(\mu +2)$ and $u=2\sqrt{2}|k|^{1+\mu /2}/(\mu +2)$^21^. Here $K_\nu (x)$ is so-called MacDonald function with the following large *x* asymptotics $K_\nu (x \rightarrow \infty )\approx (\pi /(2x))^{1/2}e^{-x}$^14^. Substitution of latter asymptotics into the above expression generates following wave function asymptotics at all admissible $\mu$’s

12 $$\begin{aligned} \chi (k \rightarrow \infty ) \sim \exp \left[ -\frac{2\sqrt{2}}{\mu +2}k^{1+\mu /2}\right] . \end{aligned}$$

Here we once more suppressed indices *nlm*. In the case of $\mu =2$ (ordinary quantum oscillator) the asymptotics (Eq. 12) reproduces well known result for “ordinary” 3D quantum oscillator^18^. It is seen that for all $0<\mu \le 2$ the asymptotics (Eq. 12) decays sufficiently fast for corresponding integrals to be convergent. Namely, in worst case of $k=0$ $\psi (k \rightarrow \infty ) \sim e^{-k\sqrt{2}}$, i.e. decays exponentially. Good localization of the wave functions in *k* space yields their localization in coordinate space by Riemann–Lebesgue lemma, see e.g.^22^. Our analysis show that at small *k* the functions *R*(*k*) have the same asymptotics $R \sim k^l$ (and accordingly $\chi \sim k^{l+1}$) as in ordinary case, corresponding to $\mu =2$^18^.

To classify the states of the 3D fractional oscillator according to the quantum numbers *nlm*, we notice that although the Eq. (9) resembles that for the ordinary 3D oscillator, it is not equivalent to it as we have the term $k^\mu$ instead of $k^2$ in the potential. The former term “spoils” the neat classification^18,19^ of the states of 3D quantum mechanical oscillator so that in the fractional case $\mu <2$ there is no obvious way to introduce the principal quantum number *n*. We recollect that for ordinary 3D oscillator $n=2n_r+l$ (see, e.g.^19^), where $n_r$ is so-called radial quantum number, defining the number of nodes of the radial function for a given *l*. That is why to classify the states of the oscillator in our 3D fractional case we begin with general consideration of a quantum particle, moving in a central force field. Namely, to apply the oscillation theorem to the radial part $\chi (k)$ in Eq. (11), we arrange the eigenenergies corresponding to a given *l* in ascending order. It had been shown^18,19^, that the lowest eigenenergy for a given *l* corresponds to a nodeless function so that we assign $n_r=0$ to this state. The next (excited) state for the same *l* has one node, i.e. $n_r=1$ etc. This means that we may classify the states of our fractional oscillator by essentially two quantum numbers - $n_r$ and *l*. To be specific, for $l=0$ (s - state; here we use the common notations of the states with the given *l*, see, e.g.^18^) we have the whole sequence $n_r=0,1,2,...$; the same is true for $l=1,2,3,..$. Introducing the principal quantum number $n=n_r+1$, we obtain following explicit classification of the states of the fractional quantum oscillator: 1*s* ($n_r=0$, hence $n=1$, $l=0$), 1*p* ($n=1$, $l=1$), 1*d* ($n=1$, $l=2$),... 2*s*, 2*p*, 2*d*,... etc. In this case, the commonly known degeneracy of the states 1*d* and 2*s*, 1*f* and 2*p* etc, inherent in “ordinary” 3D oscillator, is lifted so that all states with different $n_r$ and *l* have different energies. This classification will be used below to construct the trial functions.

To construct the above functions, we need the asymptotics at small and large *k* as well as the information about the number of nodes, i.e. the numbers $n_r$ and *l*. Specifically, in terms of functions $\chi (k)$, we have

13 $$\begin{aligned}\chi _{1s}&=A_{1s}ke^{-a_{1s}k^{1+\mu /2}},\ \chi _{2s}=A_{2s}k(a_1k+b_1) \nonumber \\&\quad \times e^{-a_{2s}k^{1+\mu /2}},\ \chi _{3s}=A_{3s}k(a_2k^2+b_2k+c_2) \nonumber \\&\quad \times e^{-a_{3s}k^{1+\mu /2}},... \end{aligned}$$

14 $$\begin{aligned}\chi _{1p}&=A_{1p}k^2e^{-a_{1p}k^{1+\mu /2}},\ \chi _{2p}=A_{2p}k(a_3k+b_3) \nonumber \\&\quad \times e^{-a_{2p}k^{1+\mu /2}},\ \chi _{3p}=A_{3p}k(a_4k^2+b_4k+c_4) \nonumber \\&\quad \times e^{-a_{3p}k^{1+\mu /2}},... \end{aligned}$$

15 $$\begin{aligned}\chi _{1d}=A_{1d}k^3e^{-a_{1d}k^{1+\mu /2}},... \end{aligned}$$

Here, $A_{nl}$ are normalization constants, found from the “1D-like” conditions

16 $$\begin{aligned}<\chi ^2(k)>=1,\ <...>=\int _0^\infty ... dk. \end{aligned}$$

Also, $a_{nl}$ are variational parameters, while the constants $a_i$, $b_i$ and $c_i$ (here $i=1-4$) are found from the orthogonality conditions

17 $$\begin{aligned} <\chi _{nl}\chi _{n'l'}>=\delta _{nn'}\delta _{ll'}, \end{aligned}$$

where $\delta _{jj'}$ is Cronecker delta. We note that the trial functions $\chi _{nl}$ for different *l* (for instance $\chi _{1p}$ and $\chi _{2d}$) are orthogonal automatically because of orthogonality of spherical harmonics^14,18,19^. That’s why the integration in Eqs. (16) and (17) runs over *k* only. This implies, that only the functions with different $n's$ (and the same *l*) should be orthogonalized. Namely, in the particular case of the above trial functions we have explicitly

18 $$\begin{aligned}<\chi _{1s}\chi _{2s}>&=<\chi _{1s}\chi _{3s}>=<\chi _{2s}\chi _{3s}>=0, \nonumber \\&\quad<\chi _{1p}\chi _{2p}>=<\chi _{1p}\chi _{3p}>=<\chi _{2p}\chi _{3p}>=0. \end{aligned}$$

The orthogonalization procedures Eqs. (17), (18) along with normalization of the functions $\chi _{nl}$ determine uniquely the coefficients $a_i$ through variational parameters $a_{nl}$.

We have explicitly

19a $$\begin{aligned}A_{1s}=\frac{(2a_{1s})^{\frac{3}{\mu +2}}\sqrt{\mu +2}}{\sqrt{2\Gamma \left( \frac{6}{\mu +2}\right) }}, \end{aligned}$$

19b $$\begin{aligned}A_{2s}&=\frac{1}{\sqrt{I_{2s}}},\ I_{2s}=\frac{2(2a_{2s})^{-\frac{10}{\mu +2}}}{\mu +2}\bigg \{a_1^2\Gamma \left( \frac{10}{\mu +2}\right) +\nonumber \\&\quad +b_1^2(2a_{2s})^{\frac{4}{\mu +2}}\Gamma \left( \frac{6}{\mu +2}\right) +\nonumber \\&\quad +2a_1b_1 (2a_{2s})^{\frac{2}{\mu +2}}\Gamma \left( \frac{8}{\mu +2}\right) \bigg \}, \end{aligned}$$

19c $$\begin{aligned}A_{1p}=\frac{(2a_{1p})^{\frac{5}{\mu +2}}\sqrt{\mu +2}}{\sqrt{2\Gamma \left( \frac{10}{\mu +2}\right) }}, \end{aligned}$$

19d $$\begin{aligned}A_{2p}&=\frac{1}{\sqrt{I_{2p}}},\ I_{2p}=\frac{2(2a_{2p})^{-\frac{14}{\mu +2}}}{\mu +2}\bigg \{a_3^2\Gamma \left( \frac{14}{\mu +2}\right) +\nonumber \\&\quad +b_3^2(2a_{2p})^{\frac{4}{\mu +2}}\Gamma \left( \frac{10}{\mu +2}\right) +\nonumber \\&\quad +2a_3b_3(2a_{2p})^{\frac{2}{\mu +2}}\Gamma \left( \frac{12}{\mu +2}\right) \bigg \}, ... \end{aligned}$$

Here $\Gamma (x)$ is the $\Gamma$-function^14^. The other normalization coefficients can be calculated, but their explicit expressions become progressively more cumbersome.

Orthogonality condition $<\chi _{1s}\chi _{2s}>=0$ yields

20 $$\begin{aligned} b_1=-\lambda a_1,\ \lambda =\frac{\Gamma \left( \frac{8}{\mu +2}\right) }{\Gamma \left( \frac{6}{\mu +2}\right) } (a_{1s}+a_{2s})^{-\frac{2}{2+\mu }}. \end{aligned}$$

Substitution of the expression (20) into the normalization coefficient $A_{2s}$ Eq. (19b) generates following explicit expression for the function $\chi _{2s}$

21 $$\begin{aligned} \chi _{2s}=\frac{k(k-\lambda )}{\sqrt{\kappa _{2s}}} e^{-a_{2s}k^{1+\mu /2}}, \end{aligned}$$

where $\lambda$ is determined by the expression (20) and

22 $$\begin{aligned}\kappa _{2s}&=\frac{2}{\mu +2}\bigg [(2a_{2s})^{-\frac{10}{\mu +2}}\Gamma \left( \frac{10}{\mu +2}\right) -2\lambda (2a_{2s})^{-\frac{8}{\mu +2}}  \nonumber \\&\quad\times \Gamma \left( \frac{8}{\mu +2}\right) +\lambda ^2 (2a_{2s})^{-\frac{6}{\mu +2}}\Gamma \left( \frac{6}{\mu +2}\right) \bigg ]. \end{aligned}$$

Now, after variational determination of the parameter $a_{1s}$ (see below), the parameter $\lambda$ (20) starts to depend on the single parameter $a_{2s}$, which can also be determined variationally. Having determined the parameters $a_{1s}$ and $a_{2s}$, we then use the orthogonality condition $<\chi _{1s}\chi _{3s}>=0$ to express both $c_2$ and $b_2$ through $a_2$. After substitution of the above found $a_{1s}$ and $a_{2s}$ into the corresponding normalization coefficient, the function $\chi _{3s}$ starts to depend on $a_{3s}$ only. Such “chain rule” can be applied to determine uniquely all higher order functions, corresponding to $l=0$ (s-state), $l=1$ (p-state) and for any arbitrary *n* and *l*.

The expressions (13)–(15) show the algorithm of construction of trial functions. This algorithm is essentially the same as that for “ordinary” case of $\mu =2$. Namely, the arbitrary trial function $\chi _{nl}$ is the product of the factors $k^{l+1}$ (small *k* asymptotics), $e^{-a_{nl}k^{1+\mu /2}}$ (large *k* asymptotics) and a polynomial of degree $n_r=n-1$ with unknown coefficients. These coefficients, in turn, are determined from the orthogonality conditions (17) by the above “chain rule”.

As usually, the variational solution of the Schrödinger equations should minimize the energies

23 $$\begin{aligned} W_{nl\mu }=\int \psi _{nlm\mu }^*(\mathbf{k})\ {\mathcal {H}_\mathbf{k}}\psi _{nlm\mu }(\mathbf{k})d^3k, \end{aligned}$$

where $\mathcal {H}_\mathbf{k}$ is the Hamiltonian (5) and $W_{nl\mu }$ are the variational approximations of the eigenenergies $E_{nl\mu }$. Normally $W_{nl\mu } \ge E_{nl\mu }$. As the radial parts $R_{nlm\mu }(k)$ of the wave functions (6) are well localized (see asymptotics (12)), the expression (23) could be rendered (by the integration by parts) to the more convenient form

24 $$\begin{aligned} W_{nl\mu }=\frac{1}{2}\int _0^\infty \bigg [\left( kR'_{nlm\mu }\right) ^2+\bigg (k^{\mu +2}+l(l+1)\bigg )R_{nlm\mu }^2\bigg ]dk, \end{aligned}$$

where $R'=dR/dk$. After substitution of the trial functions (13), (14) and (15) into the integral (24), with respect to the above “chain rule”, we find the variational parameters $a_{nl}$ from its minimization. Such a procedure can be done for all wave functions of higher excited states, giving the approximate spectrum of the operator (5). Note that the substitution of found $a_{1s}$ into $W_{1s\mu }$ (24) gives the approximate value of the ground state energy for all $\mu$, the same with, say, $a_{2p}$ gives the energy of the excited state $W_{2p\mu }$ and so on for higher eigenenergies. Although the expressions for higher excited states become extremely cumbersome (see above), the variational method permits to obtain the approximate analytical expressions for the eigenvalues and eigenfunctions of the operator (Eq. 5) for all admissible $0<\mu \le 2$.

Substitution of the trial function $\chi _{1s}$ into the integral (Eq. 24) with subsequent minimization over $a_{1s}$ yields

25 $$\begin{aligned} (a_{1s})_{min}=\frac{\sqrt{2\mu }}{2+\mu }, \end{aligned}$$

which is similar to the expression for 1D fractional oscillator^20^. Further substitution of this value to the expression (24) generates the approximate value of the ground state energy for arbitrary $\mu$

26 $$\begin{aligned} (W_{1s\mu })_{min} \approx E_{1s\mu }=\frac{1}{2}\frac{\mu +4}{\mu +2} \left( \frac{2+\mu }{2\sqrt{2\mu }}\right) ^{\frac{2\mu }{2+\mu }}\frac{\Gamma \left( \frac{2}{2+\mu }\right) }{\Gamma \left( \frac{6}{2+\mu }\right) }. \end{aligned}$$

It is seen that $(W_{1s\mu })_{min}$ gives correct value 3/2 of the ground state energy for $\mu =2$, corresponding to the ordinary quantum oscillator with the spectrum $E_n=n+3/2$ in our units. Below we shall see, that for the case $\mu =0$ all the spectrum shrinks into a single value $E_0=1/2$, which is also obtained correctly from the expression (26). The dependence (Eq. 26) will be plotted below and compared with the numerical solution.

The same procedure with $\chi _{1p\mu }$ gives that $(a_{1p\mu })_{min}=(a_{1s\mu })_{min}$, which is given by Ex. (25). The variational expression for the energy of the 1p state reads

27 $$\begin{aligned}(W_{1p\mu })_{min} \approx E_{1p\mu }=\frac{3}{2} \ \frac{8+\mu }{2+\mu }  \frac{\Gamma \left( \frac{6}{2+\mu }\right) }{\Gamma \left( \frac{10}{2+\mu }\right) }\left( \frac{2+\mu }{2\sqrt{2\mu }}\right) ^{\frac{2\mu }{2+\mu }}. \end{aligned}$$

The expression (27) shows that at $\mu =2$ we obtain the correct answer 5/2. We recollect here that the energy of “ordinary” 3D quantum oscillator is determined by the expression (8), where $n=2n_r+l$, see above. As for 1p state we have $n_r=0$ (no nodes) and $l=1$ so that $n=1$ and the energy $E_{1p}(\mu =2)=1+3/2=5/2$. At the same time, at $\mu \rightarrow 0$ we have removable divergence $\lim _{\mu \rightarrow 0}(2\mu ^{-\mu /2})=\lim _{\mu \rightarrow 0}\exp (-\mu \ln (2\mu ))=1$, while the rest of the expression (27) gives $E_{1p,\mu =0}=6\Gamma (3)/\Gamma (5)=1/2$, i.e. once more the correct answer. The dependence (27) will also be plotted below and compared with numerical results. We will see that the expressions (26) and (27) give very good approximate expressions for corresponding energies of a 3D fractional quantum oscillator for all admissible $\mu$. Within the suggested variational approach, the energy of any desired level $E_{nl\mu }$ can be evaluated analytically, although the calculations for higher excited states become very cumbersome, see above.

**Figure 2:**
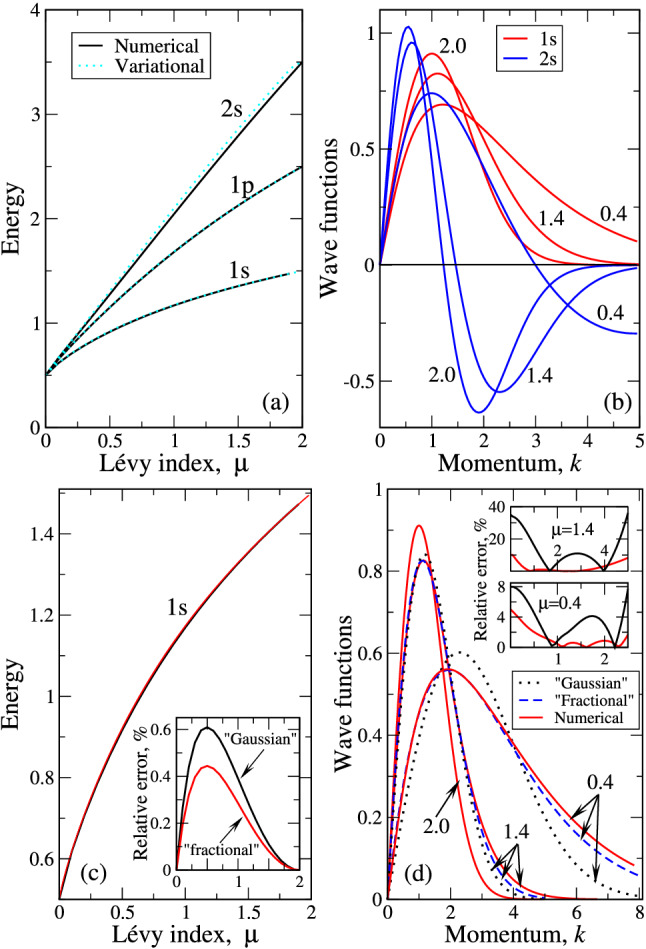
(**a**) Reports the lowest state eigenenergies (indicated near the curves) as functions of Lévy index $\mu$, calculated numerically (solid lines) and variationally (dashed lines, “fractional” functions (13)–(15)). (**b**) Displays wave functions of the lowest states, shown in the legend. The numerical and variational plots in (**b**) are similar in the scale of the plot. (**c**) The 1s state energies, calculated variationally (expressions (26) and (31)) as well as numerically. The curves are indistinguishable in the scale of the plot. Inset in (**c**) reports the “Gaussian” - numerical (black curve) and “fractional”—numerical (red curve) relative errors in the entire $\mu$ domain. (**d**) Portrays the comparison between variational (both types of the functions, shown in the legend) and numerical 1s wave functions for the same $\mu$ values, as in (**b**). Insets report the relative errors for $\mu =1.4$ and 0.4 as the wave function for $\mu =2$ is exact. Black curves correspond to “Gaussian”—numerical and red ones to “fractional”—numerical relative errors as the functions of momentum *k*. Figures near curves in (**b,d**) correspond to Lévy indices $\mu$.

## Numerical analysis

The easiest way to solve our problem numerically is to use the Eq. (11) for the function $\chi (k)$, which has only one divergency (in the centrifugal term $l(l+1)/k^2)$) at $k=0$. The numerical solution of the Eq. (11) should be augmented by the boundary conditions $\chi (0)=\chi (\infty )=0$. As usually for boundary value problems, our Eq. (11) can be solved numerically by its reduction to the eigenproblem for the corresponding finite-dimensional matrix. In this case, the energies *E* are the eigenvalues of the above matrix and the eigenvectors of the latter comprise the wave functions of the problem in momentum space. The transition to the usual coordinate space is accomplished by 3D inverse Fourier transformation. We note that the satisfactory accuracy of the numerical solution is achieved for typical matrix dimensions 10000 $\times$ 10000, which makes the task quite computer intensive.

As we can reproduce numerically the spectrum of the problem for arbitrary $\mu$, we are now in a position to compare the results of variational and numerical treatments. We begin with comparison of different classes of trial functions (see above), namely aforementioned “fractional” (13)–(15) and “Gaussian”, which we are going to construct below. As the consideration of a ground (1s in our case) state wave functions give the lower limit of the relative errors, here we consider just this function for “Gaussian” case. In terms of $\chi$ functions (10), we have

28 $$\begin{aligned} \chi _{G1s}=Ake^{-\frac{k^2}{2\sigma ^2}}, \end{aligned}$$

where “G” in lower index stands for “Gaussian”, *A* is normalization coefficient and $\sigma$ is variational parameter. We note that at $\mu =2$ the function $\chi _{G1s}$ is equivalent to $\chi _{1s}$ (see (13)) with $a_{1s}=1/(2\sigma ^2)$.

The normalization of (28) with the help of condition (16) yields

29 $$\begin{aligned} A=2\pi ^{-1/4}\sigma ^{-3/2}. \end{aligned}$$

Substitution of Eq. 28 with respect to Eq. 29 into the expression for energy (Eq. 24) with its subsequent minimization over $\sigma$ generates following minimizing wave function width

30 $$\begin{aligned} \sigma _{min}=\left( \frac{3}{2}\frac{\sqrt{\pi }}{\mu \Gamma \left( \frac{3+\mu }{2}\right) }\right) ^{\frac{1}{\mu +2}}. \end{aligned}$$

Further substitution of the ground state wave function (28), with respect to Eqs. 29 and 30, into the energy functional (24), yields the Gaussian analog of the variational ground state energy (Eq. 26)

31 $$\begin{aligned} (W_{G1s\mu })_{min} = \frac{1}{\sqrt{\pi }} \Gamma \left( \frac{3+\mu }{2}\right) \sigma _{min}^{\mu }+\frac{3}{4} (\sigma _{min})^{-2}, \end{aligned}$$

where $\sigma _{min}$ is determined by Eq. (30). At $\mu =2$, the energy $(W_{G1s\mu })_{min}$ also gives correct value 3/2 of the oscillator ground state energy. The same is valid for $\mu =0$, where $(W_{G1s\mu =0})_{min} =\Gamma (3/2)/\sqrt{\pi }=1/2$. Here we also have a removable divergency of the type $\lim _{\mu \rightarrow 0}(\mu ^{-\mu /2})=\lim _{\mu \rightarrow 0}\exp (-(\mu /2)\ln \mu )=1$ in the first term of Eq. (31).

Having both “Gaussian” (31) and “fractional” (26) and (27) expressions for variational energy levels of our 3D oscillator, we are now in a position to compare them with corresponding numerical values. Such comparison is reported in Fig. 2a,c. As is the case for the variational method, in both panels the variational curves lie higher than numerical ones as variational energy should be larger than its exact (in our case numerical) value^18^. It is seen that the agreement is a little worse for the 2s state, which has one node. For the nodeless states with $n=1$, the variational and numerical curves are indistinguishable in the scale of the plots. The quantitative error analysis for 1s state is reported in Fig. 2c. It shows that as both “Gaussian” and “Fractional” trial functions give exact results at $\mu =0$ and $\mu =2$, the relative error should be maximal somewhere inside this interval. The inset in Fig. 2c shows that the maximal relative error occurs at $\mu \approx 0.5$ and does not exceed 0.6%. Latter error occurs for “Gaussian” trial function. This is because the latter function does not take into account the actual asymptotics (Eq. 12) of the wave functions.

The selected numerical wave functions in $\mathbf{k}$-space are reported in Fig. 2b,d for different values of $\mu$. It can be checked that functions are normalized, i.e. they obey condition (16). Note that corresponding “fractional” wave functions (13) are almost indistinguishable from numerical ones in the scale of the plot in Fig. 2b. At the same time, in the Fig. 2d, we choose the vertical scale so that the (minute) differences between “fractional” (variational) and numerical 1s wave functions become visible. Panel (b) of Fig. 2 shows that functions for different $\mu$ have different decay rates, which are dictated by the asymptotics (Eq. 12). For instance, the 2s function for $\mu =0.4$ at $k=5$ is not yet achieved its final part (compare to that for $\mu =1.4$), which occurs at $k \approx 15$. For smaller $\mu$, the spatial extension of the wave functions in $\mathbf{k}$ space increases drastically. The functions for $\mu \rightarrow 0$ in $\mathbf{k}$ space are almost delocalized, which means that in coordinate space they are Dirac $\delta$-function-like. This behavior is qualitatively similar to that of 1D fractional quantum oscillator^20^ as well as for 2D^23,24^ and 3D^25^ fractional hydrogenic problems.

The same tendency is seen in Fig. 2d. Here, the “Gaussian” trial function has stronger deviation from the numerical curve, while the “fractional” one goes closer to it. This difference is more visible at $\mu =0.4$, which is close to the above value $\mu \approx 0.5$, where the relative error in the energy is maximal. As numerical and variational curves are identical for $\mu =2$ (the corresponding trial functions are exact), the relative error is reported for $\mu =1.4$ and 0.4 only. The behavior of relative error reflects the well-known fact that different classes of trial functions can give a very good accuracy for the energy, while the approximation of the numerical wave function can be much worse. We note here that the values of relative error at both $k \rightarrow 0$ and $k \rightarrow \infty$ are not that reliable as in this case we are dealing with the difference of small (in the sense of machine precision) numbers, divided by those (small) numbers. That’s why we limit ourselves by $k \approx 3$ as for $k>3$ because of latter effect, the error grows to infinity. It is seen from the insets in Fig. 2d, that in the intermediate region of *k* values, the maximal relative error of the “Gaussian” trial function is around 10%, while that for “fractional” one is less than 1%. Our analysis shows that for the “fractional” trial functions with nodes (including 2s and higher) the average error is 2–3%, the larger error around 3% occurs for the higher excited states with many nodes. This shows that the variational expressions like (13)–(15) (with respect, of course, to (19a)–(19d)) can be regarded as pretty good wave functions in $\mathbf{k}$ space. Such good variational approximation by the functions, having only one parameter may be because the energies are monotonous functions of $\mu$. That said, we can safely assert that (in the intermediate range of momenta *k*, where the error can be calculated reliably, see above) with the accuracy not higher than 3% the analytical expressions (13)–(15) approximate the corresponding numerical curves.

**Figure 3:**
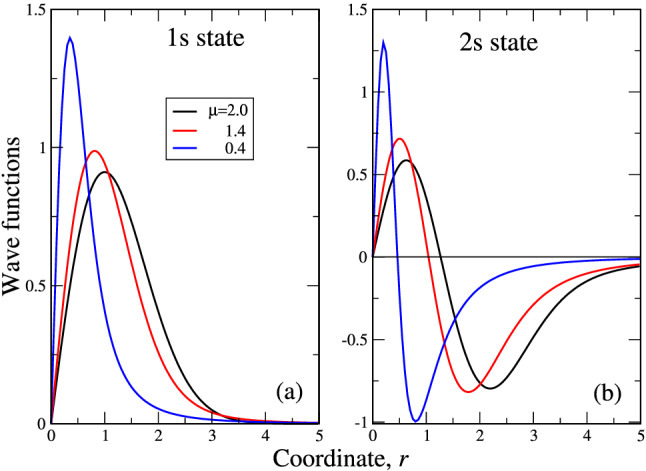
Wave functions of 1s (**a**) and 2s (**b**) states in the coordinate space. The values of $\mu$ are encoded by colors (legend in (**a**)).

One more observation is in place here. Namely, the eigenenergies in fractional case $\mu <2$ are smaller than those for “ordinary” case $\mu =2$. This means that in the system, where the substitution of conventional Laplacian by the fractional one in Schrödinger equation is admissible (like in strongly disordered solids like amorphous ones, see, e.g.^23^ for discussion), it is energetically favorable for an oscillator to “become fractional”.

As we have discussed above, the wave functions in the coordinate space can be obtained by the inverse Fourier transform. They are reported in the Fig. 3 for 1s (panel (a)) and 2s (panel (b)) states. It is seen from the Figure, that the curves are qualitatively similar to those in the momentum space. The regularities here are similar to those for 1D case^20^, i.e. the slowest decay have the functions for $\mu \rightarrow 2$ (“ordinary” quantum oscillator), while the fastest (like Dirac $\delta$, see above) have the functions for $\mu \rightarrow 0$ ($\mu =0.4$ in Fig. 3), corresponding physically to the strongest disorder. This may be one more signature of the famous Anderson localization^26^ phenomenon.

## Possible experimental applications. Outlook

The obtained spectrum of the fractional quantum 3D oscillator can be used for the calculation of arbitrary observable characteristic of real physical systems. As an illustration, here we consider the so-called oscillator strength or dipole matrix element (the matrix element of a dipole moment operator $\mathbf{d}=e\mathbf{r}$) for the transition between the energy levels with different orbital quantum numbers *l* and $l'=l \pm 1$. The latter relation stems from the selection rule, which gives zero (because of angular integration) for any combination of orbital quantum numbers, other than the latter one. The oscillator strength is important for the evaluation of such experimentally observable characteristics like the intensities of electric dipole transitions, which are the dominant effects of the interaction of a charge carrier in solids (electrons or holes) with the external electromagnetic field. One of the most important matrix elements of this type defines a transition between the states with $l=1$ and $l=0$. As here we are working in momentum space, the formula for the oscillator strength contains the matrix element $\langle a|\mathbf{k}|b \rangle$ for the transition between states *a* and *b*. In our dimensionless units, the expression for the oscillator strength reads (see, e.g.^19,27^)

32 $$\begin{aligned} \mathbf{f}_{ab}=-2\frac{|\langle a|\mathbf{k}|b \rangle |^2}{E_{a}-E_{b}}, \end{aligned}$$

where the transition occurs between states *a* (like 1*p*) and *b* (like 1*s*) with the eigenenergies $E_a$ and $E_b$ respectively. For the transition between the states 1*p* and 1*s*, the explicit expressions for matrix elements assume the form

33a $$\begin{aligned}\langle 1s|{k_x}|1p \rangle &=\int k\sin \theta \cos \varphi R_{00}(k)R_{11}(k) \times \nonumber \\&\quad \times Y_{00}Y_{1,\pm 1}(\theta ,\varphi ) d^3k, \end{aligned}$$

33b $$\begin{aligned}\langle 1s|{k_y}|1p \rangle &=\int k\sin \theta \sin \varphi R_{00}(k)R_{11}(k) \times \nonumber \\&\quad \times Y_{00}Y_{1,\pm 1}(\theta ,\varphi ) d^3k, \end{aligned}$$

33c $$\begin{aligned}\langle 1s|{k_z}|1p \rangle &=\int k\cos \theta R_{00}(k)R_{11}(k) \times \nonumber \\&\quad \times Y_{00}Y_{10}(\theta ,\varphi ) d^3k. \end{aligned}$$

where

$$\begin{aligned} \int ...d^{3}k = \int _0^\infty ...k^{2}dk \int _{0}^\pi \sin \theta d\theta \int _0^{2\pi } d\varphi . \end{aligned}$$

**Figure 4:**
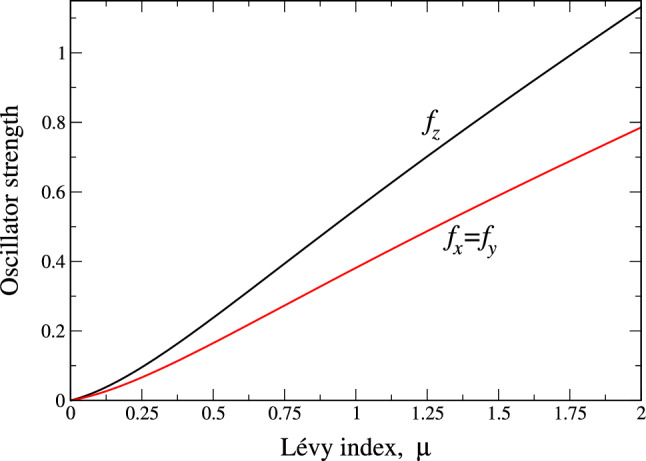
The components (shown near curves) of oscillator strength (dipole matrix element Eq. (32)) for transition between the states 1*s* and 1*p* as functions of Lévy index $\mu$. Both functions are strongly decreasing as $\mu \rightarrow 0$.

Substitution of the matrix elements (Eqs. 33a–33c) with respect to numerically calculated $R_{00}$ and $R_{11}$ wave functions into the expression (32), generates the Lévy index dependence of oscillator strength components $f_i\equiv f_{i, 1s-1p}$ ($i=x,y,z$), which is reported in Fig. 4. It is seen that the oscillator strength goes to zero in the case of strongest disorder, corresponding to $\mu =0$, see Ref.^23^ for details. We observe (see above) that as $\mu \rightarrow 0$, both $E_{1p}$ and $E_{1s}$ go to the same value (as, actually, the whole spectrum) 1/2. This means that for $\mu =0$, the denominator of the expression (32) becomes zero. Despite that, the whole expression (32) tends to zero because of much faster decay of dipole matrix elements (33a)–(33c) in this case. The origin of this stems from coordinate space, where the wave functions of all states look very much like Dirac $\delta$-functions. This means that the probability of optical transitions is much higher in relatively ordered regions, where $\mu >1$. This fact may be useful in the theoretical description of the experimental data either in polymers, where chains can vibrate, or in disordered solids. Namely, the developed formalism can be well applied to the calculations of the properties of real physical systems, where disorder (like lattice imperfections and/or impurities) influences phonon and electron spectra of a substance, leading to non-Gaussian distribution of the internal electric, magnetic and elastic fields. A compelling example is the properties of the above multiferroics^5^. The non-Gaussian statistics due to disorder and frustration plays an important role in these substances^28–30^ and it is challenging to apply the above formalism to explain their disorder (in phonon and magnon subsystems) phenomenologically in terms of introduction fractional derivatives to the corresponding Schrödinger equations. The dynamics of such “disordered phonons and magnons” can be described in terms of the above FP equation. Here, essentially the same variational approach can be used as there exist a mapping between FP and Schrödinger equation in terms of so-called Lévy–Schrödinger semigroup^31,32^. Of course, direct numerical solutions are always possible.

In this context, we also mention one more interesting physical problem regarding the onset of chaos^33^ in the systems (like semiconductors^1,2^ and trapped cold atomic gases^34^) with the joint effect of the Lorentz force, Zeeman splitting, and spin-orbit coupling^35,36^. This suggests one more generalization of our 3D fractional quantum-mechanical oscillator problem. Namely, the spin-orbit interaction term can be added to the fractional Schrödinger equation(4). In this case, the solution will be more sophisticated as the wave function will be spinor now (see, e.g.^37^) although the problem can be solved in the momentum space similar to the present case. This problem turns out to be very important for amorphous semiconductors with atomic vibrations^1,2,38^, where chaos can adversely influence possible optoelectronic, spintronic, and/or photovoltaic devices functionality.

Many physical problems can be reduced to that of a quantum harmonic oscillator. One of them is related to the theoretical studies of quark-antiquark bound states (so-called quarkonium, see, e.g. Ref.^39^), which had been a hot topic several decades ago. In the context of 1D fractional quantum oscillator, the problem had been introduced by Laskin^17^, where the 1D potentials of the form $|x|^\gamma$, $1<\gamma \le 2$ had been considered instead of quadratic one. At the same time, as the consideration was phenomenological by its nature, the linear potential had also been applied for that problem^39^. As it is well-known^18,19^, such potential in the 1D Schrödinger equation admits exact solution in terms of Airy functions^14^. In our context, such problem arises in Eq. (11) for $l=0$ and $\mu =1$. This problem had been considered by us earlier^40^ in the context of Lévy flights confinement by linear potential. The energies and wave functions of s-states (i.e. those for $l=0$) are in a very good agreement with those obtained from exact solutions in terms of Airy functions. A quantitative comparison is out of the frames of the present work. Note also, that 1D and 2D problems for the fractional Schrödinger equation in a linear potential had been considered in the paper^41^. For 1D case this paper complements both our studies of 1D fractional oscillator^20^ for the entire $\mu$ domain and those in the paper^40^ for $\mu =1$, where the exact solution in terms of Airy functions had been presented. The consideration of Cauchy distribution time evolution, made in^41^ is also complementary to our study^42^.

One more promising approach to non-relativistic quantum problems in central force potentials like $r^q$, is to suggest an approximation to the spectrum of the problem for arbitrary *q*. This has been done in the paper^43^ for the case of ordinary (i.e. non-fractional) laplacians in the Schrödinger equation. Moreover, the author^43^ was instrumental to generalize the approximation both to the case of logarithmic potential, corresponding to $(dr^q/dq)_{q=0}=\ln r$ and negative *q* like hydrogenic problems with $q=-1$. It would be interesting in future to generalize the approach^43^ to the case of fractional laplacians in corresponding Schrödinger equations.

Several one-dimensional problems (like fractional quantum harmonic oscillator and infinite quantum well as a limiting case of the potentials $|x|^p$ as $p \rightarrow \infty$) had been considered in Ref.^44^. There, the corresponding time-dependent Schrödinger equation had been solved by the matrix method. Different nonlocal operators, based on fractional Riesz derivatives had been discussed at length. Numerical and variational solutions had been presented. The variational solution of 1D fractional quantum oscillator problem had been reported in terms of Hermite and Laguerre polynomial type functions. This approach is complementary to ours in the sense that here we considered the “Gaussian” trial functions for comparison. The approach of the paper^44^ is also complementary to our 1D case, considered in Ref.^20^. The consideration of the infinite potential well problem is complementary to our work^45^, where the same problem have been solved by the explicit matrix method. Note that the matrix method had been applied in our numerical solutions of 3D (present consideration) and 1D fractional oscillators problems.

In summary, we have studied the spectral problem for a 3D fractional quantum harmonic oscillator with arbitrary Lévy index $0<\mu \le 2$. Our main supposition here is that Laskin’s construction of path integrals with Lévy measure is equivalent to “extraction” of probability density function from stochastic fractional Langevin equation and, in turn, to the assumptions made in seminal Anderson paper^26^. This permits us to assert that fractional Schrödinger equation accounts for disorder phenomenologically with Lévy index $\mu$ being the measure of the degree of disorder. The presence of potential in the system “tames” the initial Lévy distribution, making it decay faster than that in a corresponding free problem. In other words, here once more we have an interplay between the width of disorder distribution and system potential, which makes the square of the modulus of the corresponding wave function decay faster in space. This makes the problem of a fractional 3D oscillator resemblant to Anderson localization^26^ in disordered systems.

## Methods

The details of our theoretical methodology and those of working with fractional derivatives and fractional Laplacians, in particular, have been described in the sections “The spectral problem for fractional quantum harmonic oscillator” and “Variational solution. Classification of the states of 3D fractional oscillator”. The numerical solutions of boundary value problems for partial differential equation (11) have been conducted using the commercial *Mathematica* software package.

## Data Availability

The datasets used and/or analysed during the current study available from the corresponding author on reasonable request.
